# Lutetium-177 Dotatate-Induced Hemolytic Anemia and Myelodysplastic Syndrome

**DOI:** 10.7759/cureus.22392

**Published:** 2022-02-19

**Authors:** Samer Alkassis, Mohammed Ali, Abdalaziz M Awadelkarim, Eltaib Saad, Adnan Halboni, Rashid Alhusain, Saivaishnavi Kamatham, Isra Idris

**Affiliations:** 1 Internal Medicine, Wayne State University Detroit Medical Center, Detroit, USA; 2 Internal Medicine, Wayne State University Detroit Medical Center, Detroit , USA; 3 Internal Medicine, AMITA Saint Francis Hospital, Evanston, USA; 4 Internal Medicine, Wayne State, Detroit, USA; 5 Pediatrics, Woodhull Medical Center, New York, USA

**Keywords:** peptide receptor radioligand therapy, rectal neuroendocrine tumor, acute hemolytic anemia, therapy-related myelodysplastic syndrome, lutetium-177 dotatate

## Abstract

Lutetium-177 (^177^Lu) dotatate is a type of peptide receptor radioligand therapy (PRRT) using radiolabeled somatostatin for patients with progressive somatostatin receptor-positive gastrointestinal neuroendocrine tumors. While cases of therapy-related myeloid neoplasms (t-MN) have been described as a consequence of ^177 ^Lu dotatate, there are no reports of hemolytic anemia associated with therapy. We present a case of a 68-year-old woman with metastatic low-grade neuroendocrine tumor who presented four weeks after the second dose of^ 177^Lu dotatate with progressive fatigue and dyspnea. Laboratory workup was remarkable for hemolytic anemia. Lutetium-177 dotatate-induced hemolysis was suspected after ruling out other causes. Corticosteroid treatment was initiated with improvement in hemoglobin, and dose-reduced PRRT was planned upon discharge. Six months into the treatment course of ^177^Lu dotatate, macrocytic anemia was noticed on routine follow-up with normal vitamin B12 and folic acid levels. A bone marrow biopsy was done, revealing myelodysplastic syndrome (MDS) features. Given the temporal relationship between drug introduction and the objective findings, early-onset ^177^Lu dotatate-induced MDS was diagnosed with a plan for close hematologic follow-up. Myelodysplastic syndrome should be suspected when megaloblastic anemia develops in patients with previous ^177^Lu dotatate therapy. The latency period between initial treatment and MDS diagnosis reported in the literature ranges between 15 months to seven years. Apart from the unusually early onset of MDS, what is unique about our case is the development of hemolytic anemia after administration of PRRT. The clinical course and the brisk response to steroid therapy, suggest other mechanisms of PRRT toxicity besides DNA breaks, genetic mutations, and myelosuppression by an immune-mediated component that likely plays a role in ^177^Lu dotatate toxicity. Further investigation and monitoring are needed to identify the frequency of such adverse events and the pathophysiology of their occurrence.

## Introduction

Lutetium-177 (^177^Lu) dotatate, commonly known as Lutathera®, is a peptide receptor radioligand therapy (PRRT) using radiolabeled somatostatin. On January 26, 2018, the Food and Drug Administration (FDA) approved its use for patients with somatostatin receptor-positive gastroenteropancreatic neuroendocrine tumours (GEP-NETs), who have progressed during somatostatin analog therapy [1]. Lutetium is a lower energy beta-emitting radionuclide. Exposure to high doses of radiation occurs by selectively binding to tumor cells with limited toxicity on normal tissue [2]. Despite this fact, hematologic adverse events have been commonly reported - myelosuppression was reported as a side effect during drug development. However, persistent hematologic dysfunction, defined as myelodysplastic syndrome (MDS), is uncommon [3-5]. The latency period between initial treatment with ^177^Lu dotatate and MDS diagnosis ranges between 15 months to seven years. While cases of therapy-related myeloid neoplasms (t-MN) have been described in the literature, there are no reports of hemolytic anemia. Herein, we present a case of ^177^Lu dotatate-induced hemolysis and MDS that developed as an early and delayed adverse effect of therapy, respectively.

## Case presentation

**Figure 1:**
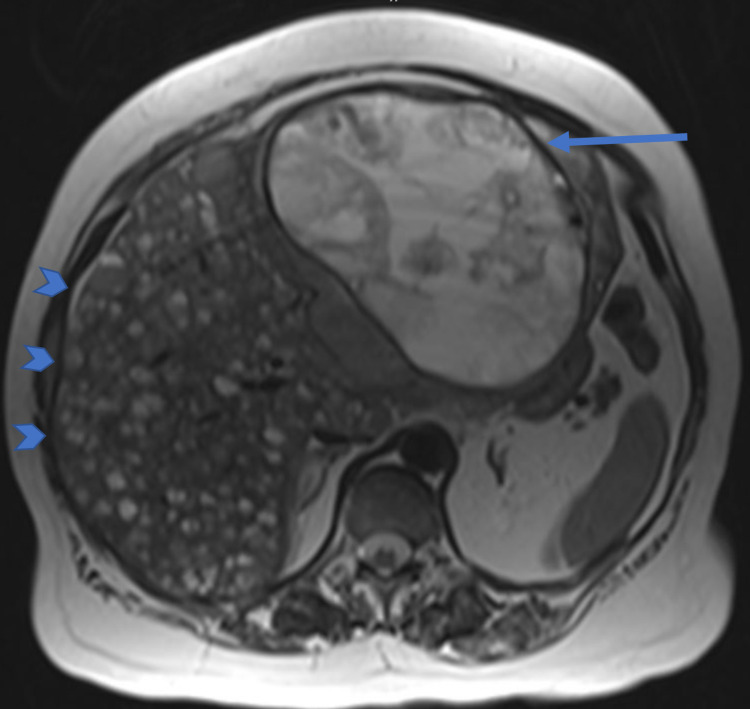
MRI of the abdomen showing an intrinsic T1 hyperintense lesion consistent with a large 14.9 x 14.0 cm complex necrotic mass in the left hepatic lobe (blue arrow), with innumerable small metastatic lesions throughout the liver parenchyma (blue arrowheads).

A 68-year-old woman with a past medical history of essential hypertension (amlodipine 10 mg) and cholelithiasis (status post cholecystectomy) was found to have a rectal polyp during a routine colonoscopy with a biopsy revealing a low-grade neuroendocrine tumor. Endo-mucosal resection of the polyp was attempted a few months later. However, invasion of the muscularis propria was noted. Therefore, the procedure was aborted. A CT scan of the abdomen and pelvis was done for further evaluation showing a large complex mass in the left lobe of the liver. This was followed by an MRI of the bdomen, which showed a cystic liver lesion with rim enhancement, and multiple additional smaller lesions (Figure 1). She was started on octreotide 20 mg every four weeks. However, a repeat CT scan showed disease progression after five months of therapy. The dose of octreotide was increased to 30 mg, and she was referred to our institution for further evaluation. Given the lack of response to octreotide, she was deemed a candidate for palliative ^177^Lu dotatate, with a plan for a total course of four doses to be administered every eight weeks. The first dose was tolerated without complications. However, she was admitted to the hospital four weeks after the second dose with malaise, progressive fatigue, and dyspnea. 

Laboratory workup on presentation revealed normocytic anemia (6.2 g/dL from a baseline of 11 g/dL [Normal range 12.1-15.0 g/dL]) and grade II thrombocytopenia. Further hematologic workup was significant for low haptoglobin, high lactate dehydrogenase (LDH), and indirect hyperbilirubinemia. Refer to Table 1 for complete hematologic indices with their reference ranges. Peripheral blood smear revealed anisocytosis, burr cells, and very few schistocytes. Hemolytic workup, including direct antiglobulin test; a disintegrin and metalloproteinase with a thrombospondin type 1 motif, member 13 (ADAMTS 13); cold agglutinin; and viral panel screening, was negative. The patient required one unit of packed red blood cell transfusion. Lutetium-177 dotatate-induced hemolysis was suspected after consultation with the hematology team. The patient received a six-day course of prednisone 80 mg daily with improvement in hemoglobin close to her baseline. However, the plan upon discharge was to continue with dose-reduced PRRT; she received two additional doses of ^177^Lu dotatate to complete four doses.

**Table 1:** Laboratory workup at the time of presentation with their reference ranges ADAMTS13: A disintegrin and metalloproteinase with a thrombospondin type 1 motif, member 13

Laboratory test	Patient’s result	Reference range
White Cells Count (WCC)	6.5 k/ 𝜇L	3.3- 10.7 k/ 𝜇L
Absolute Neutrophil Count	4.7 k/𝜇L	1.6- 7.2 k/𝜇L 𝜇L
Hemoglobin	6.2 g/dl (Baseline 11 g/dl)	12.1- 15.0 g/dL
Hematocrit	19.9%	34.4-44.2%
Mean Corpuscular Volume (MCV)	96 fL	80- 100 fL
Absolute reticulocyte count	123,500𝜇L	20,000-100,000𝜇L
Reticulocyte%	5.5%	0.5-2.0%
Platelets	99 k/ 𝜇L	150–400 k/ 𝜇L
Mean Platelets Volume	10.1 fL	7-12 fL
Total Bilirubin	3.47 mg/dL	<1.5 mg/dl
Indirect Bilirubin	1.52 mg/dL	0.0-0.80 mg/dL
Direct Bilirubin	1.95 mg/dL	0.03-0.18 mg/dL
Aspartate Transference (AST)	20 units/L	7-52 units/L
Alanine Aminotransferase (ALT)	39 units/L	13-39 units/L
Alkaline Phosphatase (ALP)	170 units/L	50-142 units/L
Lactate Dehydrogenase (LDH)	319 unit/L	140-217 unit/L
Haptoglobin	<30 mg/dL	9.0 -25.0 mg/dL
Fibrin Monomers	negative	negative
Fibrinogen	378 mg/dl	186-466 mg/dl
International Normalized Ratio (INR)	1.03	0.90 -1.13
Activated Partial Thromboplastin Time (APTT)	27.6	23.1-33.1
Cold Agglutinins	< 1:32	< 1:32
ADAMTS13	69%	75 – 117%

**Figure 2:**
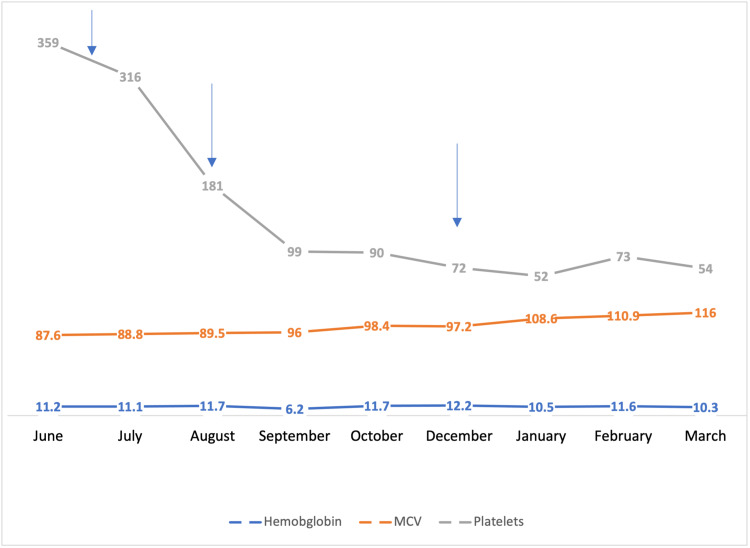
Graph depicting hemoglobin [g/dL], mean corpuscular volume (MCV) [fL], and platelets [k/𝜇L] during and after treatment with lutetium-177 dotatate

Five weeks after the last dose of PRRT, macrocytic anemia was demonstrated on regular follow-up with persistent thrombocytopenia (Figure 2). Vitamin B12 and folic acid were within normal limits. Repeat peripheral smear showed anisopoikilocytosis consisting of ovalocytes, acanthocytes, burr cells. Further investigation with 13bone marrow biopsy was obtained, revealing dyserythropoiesis with mild dysmegakaryopoiesis suggestive of MDS. Cytogenetics revealed a uniform pattern of a normal female karyotype with no consistent numerical or structural clonal aberration. The fluorescence in situ hybridization (FISH) panel was also negative. Given the temporal relationship between drug introduction and the objective findings, ^177^Lu dotatate-induced MDS was diagnosed. The plan was to continue to follow up in the hematology clinic and to check blood count regularly. 

## Discussion

Lutetium-177 dotatate is a combination of the radionuclide ^177^Lu with the somatostatin analog DOTA-TATE. Single- and double-stranded DNA breaks are induced by ionizing radiation specifically to somatostatin-receptors-positive tumor cells, leading to tumor cell death [6]. As a result of DNA breaks, errors in the repair mechanism occur and contribute to genetic mutations [4]. This also could be the potential pathogenesis of ^177^Lu dotatate-induced hematologic toxicity. 

The incidence of therapy-related myeloid neoplasms (t-MN), including MDS, acute leukemia, and myeloproliferative disorder, has been reported to be 2.61% in a recent systematic review [7]. In a clinical trial involving 807 patients with neuroendocrine neoplasm treated with PRRT, MDS and acute myeloid leukemia (AML) occurred in 2.35% and 1.1% of patients, respectively [4]. Another study of 274 patients who underwent PRRT with 177Lu for gastro-entero-pancreatic neuroendocrine tumors (GEP-NETs) showed hematopoietic neoplasm in 2.9% of the patients, and bone marrow failure in 1.1% who developed bone marrow failure characterized by cytopenia. In a small study in France, the incidence of MDS/AML was high (20%) in patients with GEP-NETs treated with PRRT following treatment with alkylating chemotherapy [8]. 

As demonstrated above, a comprehensive literature review yields multiple reports that describe t-MN as a consequence of PRRT. Myelodysplastic syndrome should be suspected when megaloblastic anemia develops in patients with previous ^177^Lu dotatate therapy. The latency period between initial treatment and MDS diagnosis reported in the literature ranges between 15 months to seven years [3]. However, what is unique about our case is the early onset of MDS (six months into treatment course) and the development of hemolytic anemia after administration of PRRT. 

The brisk response to corticosteroids and the return of hemoglobin to baseline values are strongly supportive of immune-mediated hemolytic anemia. The absence of laboratory evidence of other causes of hemolysis increased clinical suspicion of PRRT as the etiology of the observed hemolysis. However, Coombs-negative autoimmune hemolytic anemia has been described in association with MDS. Additionally, the lack of platelet count response to steroids hints that thrombocytopenia was likely not immune-mediated and could be explained by either bone marrow toxicity of PRRT therapy or secondary to an underlying MDS that was undiagnosed at the time of presentation [6-8]. However, the later absence of significant anemia at the time of MDS diagnosis, the relatively low mean platelet volume, and the marginally elevated mean corpuscular volume (MCV) on presentation don't support MDS as the underlying etiology for the immune-mediated hemolytic anemia observed in our patient.

To our knowledge, ^177^Lu dotatate-induced hemolysis has not been described before in the literature. Furthermore, the nature of this adverse event suggests the presence of other mechanisms of PRRP toxicity besides DNA breaks, genetic mutations, and myelosuppression [1,2]. An immune-mediated component likely plays a role given the improvement of hemoglobin after corticosteroid administration and the return of hemoglobin to baseline levels at the time of MDS diagnosis.

## Conclusions

Myelodysplastic syndrome should be suspected when megaloblastic anemia develops in patients with previous^ 177^Lu dotatate therapy. Immune-mediated hemolytic anemia is a potential adverse effect of PRRT therapy. Further investigations are needed to identify the frequency of such adverse events and the pathophysiology of their occurrence.
